# Therapeutic Effects of Pharmacological Modulation of Serotonin Brain System in Human Patients and Animal Models of Fragile X Syndrome

**DOI:** 10.3390/ijms26062495

**Published:** 2025-03-11

**Authors:** Lucia Ciranna, Lara Costa

**Affiliations:** 1 Department of Biomedical and Biotechnological Sciences, University of Catania, 95123 Catania, Italy; 2 Department of Clinical and Experimental Medicine, University of Messina, 98125 Messina, Italy; lcosta@unime.it

**Keywords:** Fragile X syndrome, serotonin, SSRI, 5-HT_1A_, 5-HT_2A_, 5-HT_5A_, 5-HT_7_

## Abstract

The brain serotonin (5-HT) system modulates glutamatergic and GABAergic transmission in almost every brain area, crucially regulating mood, food intake, body temperature, pain, hormone secretion, learning and memory. Previous studies suggest a disruption of the brain 5-HT system in Fragile X Syndrome, with abnormal activity of the 5-HT transporter leading to altered 5-HT brain levels. We provide an update on therapeutic effects exerted by drugs modulating serotonergic transmission on Fragile X patients and animal models. The enhancement of serotonergic transmission using Selective Serotonin Reuptake Inhibitors (SSRIs) corrected mood disorders and language deficits in Fragile X patients. In *Fmr1* KO mice, a model of Fragile X Syndrome, selective 5-HT_7_ receptor agonists rescued synaptic plasticity, memory and stereotyped behavior. In addition, drugs specifically acting on 5-HT_1A_, 5-HT_2_ and 5-HT_5_ receptor subtypes were able to correct, respectively, epilepsy, learning deficits and hyperactivity in different Fragile X animal models. In conclusion, the SSRI treatment of Fragile X patients improves mood and language; in parallel, studies on animal models suggest that compounds selectively acting on distinct 5-HT receptor subtypes might provide a targeted correction of other Fragile X phenotypes, and thus should be further tested in clinical trials for future therapy.

## 1. Introduction

### 1.1. Physiological Role of Serotonin

Serotonin (5-HT) is a neurotransmitter and neuromodulator widely distributed in the central nervous system and in peripheral tissues, particularly in the gut and in blood platelets. In the brain, 5-HT is produced by neurons located in raphe nuclei and projected to cortical areas, limbic structures, the hippocampus, the hypothalamus, the cerebellum and the spinal cord, by which 5-HT regulates mood, cognition, hormone production, food intake, body temperature, the sleep–wake cycle and pain transmission [1]. In addition, 5-HT plays a crucial role in neurite outgrowth and synapse formation during brain development [2], and a disruption of 5-HT transmission at early developmental stages is a recognized cause of autism [1,3]. 5-HT exerts its effects on target neurons by the activation of many different subtypes of receptors, grouped into seven families, from 5-HT_1_ to5-HT_7_ [4].

### 1.2. Serotonin Receptors

Almost all 5-HT receptors are G-protein-coupled metabotropic receptors, with the exception of the 5-HT_3_ type, which belongs to the superfamily of ligand-gated ion channels. 5-HT_1_ receptors are negatively coupled to adenylate cyclase and include five subtypes named 5-HT_1A_, 5-HT_1B_, 5-HT_1D_, 5-ht_1E_ and 5-HT_1F_ (lower case appellations were attributed to 5-HT receptors that were cloned but not identified in native tissues). 5-HT_1A_ receptors are widely distributed in the brain, particularly in limbic areas and in the hippocampus; in raphe nuclei they are expressed on the soma and dendrites of serotonergic neurons, where they function as autoreceptors, inhibiting cell firing and 5-HT release in target brain areas. The 5-HT_1B_ and 5-HT_1D_ receptors are highly expressed in basal ganglia; these receptors also exert vascular effects and are the target of sumatryptan and related antimigraine drugs. The subtype initially named 5-HT_1C_ was later found to be coupled to phosphoinositide signaling, and was thus renamed 5-HT_2C_ and included in the 5-HT_2_ receptor family.

The 5-HT_2_ receptors include 5-HT_2A_, 5-HT_2B_ and 5-HT_2C_ subtypes, which are coupled to Gq and stimulate phospholipase C, inositol 3-phosphate signaling and intracellular calcium release [4]. 5-HT_2A_ receptors are highly expressed in the cerebral cortex, in the olfactory bulb and in many brainstem nuclei (motor trigeminal, facial and hypoglossal); intermediate levels were found in limbic areas and in basal ganglia, and they have very little expression in the hippocampus and no expression in the cerebellum and thalamus. In the brain, 5-HT_2A_ receptor activation induces depolarizing effects, whereas in peripheral tissues they stimulate smooth muscle contraction. 5-HT_2B_ receptors are expressed in many peripheral tissues; their brain expression is strictly limited to the cerebellum, lateral septum, hypothalamus and amygdala, and they seem to play a role in anxiety. 5-HT_2C_ receptors (initially identified in the choroid plexus and named 5-HT_1C_) are expressed in the hippocampus, in limbic structures, in some thalamic nuclei and in the basal ganglia of rodents, and their malfunction might be involved in affective disorders.

5-HT_3_ receptors (including two subtypes, 5-HT_3A_ and 5-HT_3B_) are ligand-gated cation channels mediating fast depolarization; they are located on neurons of the dorsal vagal complex responsible for the vomiting reflex, which accounts for the antiemetic action of 5-HT_3_ receptor antagonists [4]. In addition, 5-HT_3_ receptors are expressed in mouse and rat hippocampus GABAergic interneurons and play a role in synaptic plasticity [5,6].

5-HT_4_ receptors were identified in mouse colliculi neurons and in guinea pig brains for their ability to stimulate adenylate cyclase activity and cyclic adenosine monophosphate (cAMP) production [7,8]. They are expressed in peripheral tissues, including the heart and the intestines, and in several brain regions, particularly in the limbic system, hippocampus and brain cortex. Several studies show that the activation of 5-HT_4_ receptors exerts pro-cognitive effects; thus, these receptors have become a target in cognition deficits [9,10].

5-HT_5_ receptors were cloned in 1994, but their expression in native tissues remained uncertain until recently and their identification was difficult due to the lack of selective ligands. Two receptor subtypes, 5-HT_5A_ and 5-HT_5B_, have been identified in mice and rats, whereas in the human brain only the 5-HT_5A_ subtype is expressed. Their predominant signaling mechanism is the inhibition of adenylate cyclase activity through Gi/o proteins. Interestingly, 5-HT_5_ receptors are selectively expressed in the brain, particularly in the hippocampus and frontal cortex; their physiological role, still to be clarified, seems to be related to the control of the circadian rhythm, mood and learning [11].

5-HT_6_ receptors, similar to 5-HT_5_, are brain-specific: they have been localized in rat and human striatum, amygdala, hippocampus, cortex and olfactory tubercle. 5-HT_6_ receptors stimulate adenylate cyclase activity; they modulate cholinergic and monoaminergic transmission, and their physiological function, still to be clarified, is related to mood and learning [4,12].

5-HT_7_ receptors were cloned in 1993 by three distinct laboratories [13,14,15]. In rat and mouse brains, 5-HT_7_ receptors are highly expressed in the thalamus, hypothalamus and hippocampus, with lower amounts in the cerebral cortex, amygdala, striatum, cerebellum and spinal cord (reviewed by [16]). In the human brain, 5-HT_7_ receptors were found in the same brain areas as in rat and mouse models, with high expression levels in the thalamus, dorsal raphe, hippocampus and hypothalamus [17,18]. In addition, the human brain contains high levels of 5-HT_7_ receptors in the caudate nucleus, putamen and substantia nigra [17]. 5-HT_7_ receptors are metabotropic receptors coupled to G_s,_ and they induce adenylate cyclase stimulation, cAMP formation and the activation of several kinases, including protein kinase A (PKA) [13,15,19], extracellular signal-regulated kinase (ERK) [20,21,22] and the kinase Akt (also known as protein kinase B) [23,24]. Interestingly, 5-HT_7_ receptors are also linked to G_12_ [25], a heterotrimeric G-protein-modulating “small” monomeric GTPases [26]. Through the G12-dependent activation of the small GTPases RhoA and Cdc42, 5-HT_7_ receptor activation regulates gene transcription, neuronal morphology, neuronal excitability and synaptic plasticity [25,27,28]. 5-HT_7_ receptors play a crucial role in neurite and dendrite outgrowth during development [28,29], control sleep and thermoregulation [27] and exert pro-cognitive effects [30,31,32]. In our laboratory, we found that 5-HT_7_ receptors modulate synaptic transmission and plasticity in the hippocampus of wild-type mice and rescue defects in synaptic plasticity, learning and behavior in *Fmr1* KO mice, a model of Fragile X Syndrome, and are thus a promising target for the future therapy of this disease (see below).

### 1.3. Fragile X Syndrome

Fragile X Syndrome is a genetic form of intellectual disability affecting 1/4000 males and 1/8000 females, caused by the silencing of the FMR1 gene, located on the X chromosome, coding for Fragile X Messenger Ribonucleoprotein Protein (FMRP) [33,34]. FMRP is an mRNA-binding protein rapidly produced in neuronal dendrites following the activation of metabotropic glutamate receptors (mGluRs) [35], and plays a crucial role as a translation modulator of a large number of synaptic proteins [36,37]. In Fragile X Syndrome, the silencing of the *FMR1* gene leads to the reduced or absent production of FMRP, causing a disruption of synaptic structure and function: abnormally long, thin and immature dendritic spines have been observed in the brain cortex of Fragile X patients [38] and of *Fmr1* gene knockout (*Fmr1* KO) mice, a model of this disease [39]. Electrophysiology studies on *Fmr1* KO mice hippocampi first identified a peculiar alteration of synaptic plasticity: in particular, long-term depression induced by metabotropic glutamate receptors (mGluR-LTD) was abnormally enhanced [40], leading to the “mGluR theory” of Fragile X Syndrome [41]. In addition, several alterations of synaptic transmission were later discovered, including disrupted mGluR coupling to intracellular signaling [42], altered cell-surface receptor mobility and a lack of mGluR-LTD or NMDA-mediated synaptic currents [43], a reduced NMDA/AMPA ratio [43,44,45], altered NMDA-dependent plasticity [46,47] and reduced GABAergic inhibitory transmission [48,49,50,51]. 

Defects in synaptic transmission and plasticity in turn impair learning, memory, mood and behavior, both in human Fragile X patients [52] and in animal models of the disease [53]. The most serious Fragile X symptoms (with variable severity in different patients) include cognitive deficits, epilepsy, attention deficit and hyperactivity disorder, anxiety and autistic behavior [52]. Since mood and cognition are crucially regulated by 5-HT in physiological conditions, the modulation of serotonergic transmission might improve impaired learning and behavioral functions in Fragile X Syndrome. In the following paragraphs, we will review the literature showing that 5-HT brain functions are probably altered in Fragile X Syndrome and that the pharmacological modulation of 5-HT neurotransmission can correct many symptoms of the disease.

## 2. Serotonin Dysregulation in Fragile X Patients and Therapeutic Effects of Selective Serotonin Reuptake Inhibitors

Brain 5-HT levels and the amount of expression of 5-HT receptors have not been measured in Fragile X patients. A dysregulation of the brain 5-HT system during development is well known in non-syndromic forms of autism, and thus might also occur in Fragile X Syndrome [54].

In young Fragile X patients, polymorphisms on the 5-HT transporter (5-HTT or SERT) gene were found to be highly related to aggressive and autistic behavior. In particular, Fragile X patients showing the highest level of aggressive, self-injuring and repetitive behavior were homozygous for a high-transcribing long form of the gene, leading to an enhanced expression of 5-HTT and a higher re-uptake of serotonin. On the other side, patients homozygous for a short genotype, with a lower expression of 5-HTT and a less efficient serotonin re-uptake, showed the lowest levels of aggressive and self-destructive behavior [55].

A very recent work shows reduced 5-HT levels in the striatum of young adult *Fmr1* KO mice [56]. In *Fmr1* KO mice of early post-natal age, the expression level of the mRNA for 5-HTT (also named SERT) was reduced in their thalamic nuclei, suggesting that 5-HT levels were altered; this might account for the plasticity defects of *Fmr1* KO mice during the critical period of development [57]. Another work shows that, in physiological conditions, FMRP interacts with 5-HTT, suggesting that in Fragile X Syndrome the lack of FMRP alters 5-HTT expression and/or activity [58].

Selective Serotonin Reuptake Inhibitors (SSRIs), enhancing 5-HT levels in the synaptic cleft, are very effective antidepressant and anti-anxiety drugs, and are often prescribed to Fragile X patients to reduce anxiety and depression [54]. In addition to mood disorders, SSRIs also correct other symptoms (Table 1): a case report on a very young Fragile X patient (3 years old) with a delay in language development showed a significant improvement of speech ability, described as an “explosion of verbalization”, following treatment with sertraline, an SSRI [59]. This observation was later confirmed by a clinical trial on very young children (2 to 6 years old) with Fragile X Syndrome, in which 6 months of treatment with a low dose of sertraline improved language expression, particularly in children with concomitant autistic features. Sertraline treatment also improved the visual perception and fine motor skills of children with Fragile X [60]. An improvement in language expression by SSRI treatment was observed specifically in Fragile X patients and not in non-syndromic autistic patients [61].

Some variability in the responsiveness of different Fragile X patients to SSRIs was found to be related to polymorphisms in genes involved in serotonergic transmission, including the 5-HTT gene [62].

Taken together, all these studies indicate that enhancing brain serotonergic transmission by SSRI treatment improves the mood and language of Fragile X patients, particularly when treatment is started at a young age during the critical period of brain development.

**Table 1:** Effects of drugs modulating serotonergic transmission in Fragile X patients and animal models.

Pharmacological Category	Drug	Experimental Model	Effects	References
Selective Serotonin Reuptake Inhibitor (SSRI)	Sertraline (2.5 mg/day) Sertraline (20 mg/day)	Fragile X patients, one boy (3 years old) one girl (7 years old).	Improvement of speech ability. Reduced anxiety	[59]
SSRI	Sertraline (2.5–5 mg/day)	Fragile X patients (2–6 years old, 48 males and 9 females).	Improvement of language, visual perception and fine motor skills.	[60]
5-HT_1A_ agonist	FPT (mixed agonist of 5-HT_1_, 5-HT_2C_ and 5-HT_7_ receptors), 5.6 mg/kg.	*Fmr1* KO mice, (males and females)	Prevention of audiogenic seizures; increase of social interaction; anxiolytic effects.	[63]
5-HT_1A_ agonist	FPT (5.6 mg/kg)	*Fmr1* KO mice (males and females)	Rescue of electroencephalogram activity	[64]
5-HT_1A_ agonist	NLX-101 (1.2–2.4 mg/Kg)	*Fmr1* KO mice (males and females)	Reduction of audiogenic seizures	[65]
5-HT_1A_ agonist	NLX-112 (1.0–2.5 mg/Kg)	*Fmr1* KO mice (males and females)	Prevention of audiogenic seizures	[66]
5-HT_1A_ agonist	Eltoprazine (1 mM for 30 min)	FXS Drosophila model (sex not indicated)	Rescue of abnormal mitochondrial function; rescue of locomotor activity	[67]
5-HT_2A_ antagonist	MDL11939 (1 μM for electrophysiology; 1 mg/Kg for behavioral tests)	*Fmr1* KO mice (males and females)	Rescue of synaptic plasticity (GluA1 synaptic delivery); partial rescue of learning deficits	[68]
5-HT_2B_ agonist	BW723C86 (1 μM for electrophysiology; 5 mg/Kg for behavioral tests)	*Fmr1* KO mice (males and females)	Rescue of synaptic plasticity (GluA1 synaptic delivery); partial rescue of learning deficits	[68]
5-HT_5A_ antagonist	ASP5736 (0.01–0.1 mg/kg)	*Fmr1* KO rats (males)	Correction of hyperactivity, abnormal sensory motor gating and learning deficits	[69]
5-HT_7_ agonist	5-HT (10 μM); 8-OH-DPAT (100 nM); LP-211 (10 nM); BA-10 (10 nM)	*Fmr1* KO mice (males and females)	Rescue of synaptic plasticity (mGluR-LTD)	[70,71]
5-HT_7_ agonist	LP-211 (10 nM)	*Fmr1* KO mice (males and females)	Rescue of synaptic plasticity, learning and stereotyped behavior.	[72]
5-HT_7_ agonist	LP-211 (3 mg/Kg)	*Fmr1* KO mice (males)	Rescue of stereotyped behavior	[73]

## 3. Selective Agonists of 5-HT_7_ Receptors Rescued Synaptic Plasticity, Learning Deficits and Autistic Behavior in *Fmr1* KO Mice

**Figure 1:**
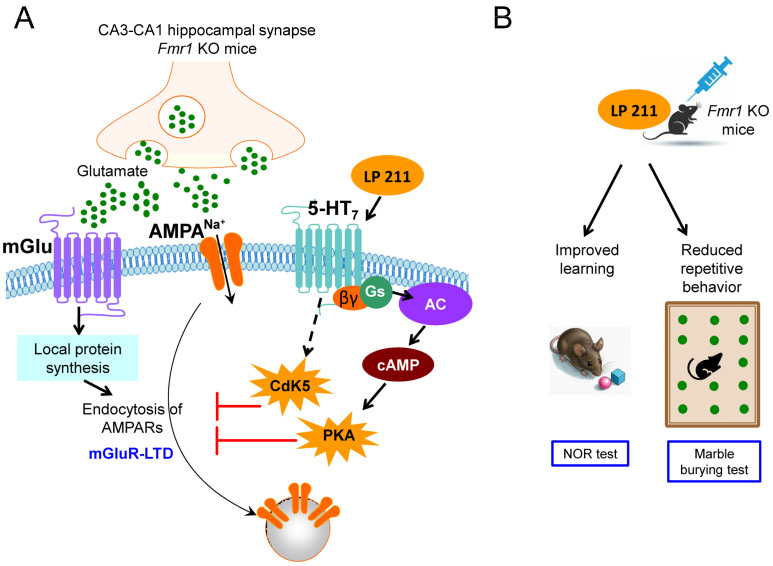
The activation of 5-HT_7_ receptors corrected exaggerated mGluR-LTD, improved learning and reduced stereotyped behavior of *Fmr1* KO mice. (**A**) In mouse hippocampal synapses between the CA3 and CA1 pyramidal neurons, the activation of group I metabotropic glutamate receptors induces long-term depression (mGluR-LTD), mediated by local protein synthesis, the activation of phosphatases and the endocytosis of AMPA glutamate receptors. In *Fmr1* KO slices, the amount of mGluR-LTD was abnormally enhanced with respect to wild-type mice and was reduced by the application of LP-211, a selective 5-HT_7_ receptor agonist: the activation of 5-HT_7_ receptors prevented the mGluR-induced endocytosis of AMPA receptors and rescued mGluR-LTD to normal levels [70]. The effect of LP-211 was mediated by the activation of adenylate cyclase (AC), cAMP formation and the stimulation of protein kinase A (PKA) [72]. The 5-HT_7_ receptor-mediated rescue of mGluR-LTD also involved cyclin-dependent kinase 5, Cdk5 [81]; the intracellular pathway leading to Cdk5 activation is still to be clarified (dotted line). (**B**) The in vivo intraperitoneal injection of LP-211 into *Fmr1* KO mice improved memory in Novel Object Recognition (NOR) test and reduced repetitive behavior, as measured by the marble burying test [81]. Parts of images from the Motifolio drawing toolkit (www.motifolio.com) were utilized in this figure.

As above mentioned, 5-HT_7_ receptors are expressed in human brain areas involved in learning, particularly in the thalamus, hypothalamus, amygdala and hippocampus [74], and several reports show that 5-HT_7_ receptor activation exerts pro-cognitive effects in experimental animal models [32]. In our laboratory, we initially observed the short-term effects of two distinct 5-HT receptors on basal glutamatergic transmission in the hippocampus of wild-type mice: AMPA receptor-mediated synaptic transmission was reduced by the activation of 5-HT_1A_ receptors by a pre-synaptic mechanism, and was enhanced at a post-synaptic level by the activation of 5-HT_7_ receptors [75]. Therefore, we decided to study the effects of 5-HT receptors in *Fmr1* KO mouse hippocampi, in which long-term depression mediated by metabotropic glutamate receptors (mGluR-LTD) is exaggerated due to the excessive removal of AMPA receptors [40]. We found that the activation of 5-HT_7_ receptors reversed hippocampal mGluR-LTD in both wild-type and *Fmr1* KO mice, in which this form of plasticity is abnormally enhanced: the brief application (5 min) of a 5-HT_7_ receptor agonist exerted a long-lasting reversal of mGluR-LTD that was still observed over 45 min after LTD induction (Figure 1A and Table 1). This effect of 5-HT_7_ receptors was exerted at a post-synaptic level by preventing the mGluR-induced membrane removal of AMPA receptors [70]. The reversal of mGluR-LTD by 5-HT_7_ receptors was mediated by adenylate cyclase activation and protein kinase A stimulation (Figure 1A) [72]. This result is in line with the “cAMP theory of Fragile X Syndrome”, which is based on several observations: cAMP production is reduced in the blood platelets of Fragile X patients [76,77], in the brain of drosophila and mouse Fragile X models and in neural precursor cells from human Fragile X fetal tissues [78]; phosphodiesterase 2a (an enzyme breaking down cAMP) is one of the main targets of physiological FMRP inhibitory control [79], and is thus overactive in *Fmr1* KO mouse neurons, leading to reduced cAMP levels [80].

We found that the 5-HT_7_ receptor-mediated reversal of mGluR-LTD also involved activation of cyclin-dependent kinase 5 (Cdk5; Figure 1A) [81], a kinase that plays a physiological role in brain development and function [82] and is disrupted in several disorders of the central nervous system [83]. Interestingly, Cdk5 expression was found to be reduced in the hippocampus of *Fmr1* KO mice [84]. Cdk5 downregulation has also been associated with epilepsy [85] and attention deficit and hyperactivity disorder (ADHD) [86], both frequent symptoms in Fragile X patients. Concerning the 5-HT_7_ receptor-mediated rescue of mGluR-LTD that we described in *Fmr1* KO mice, the relationship between cAMP formation and Cdk5 activation by 5-HT_7_ receptors needs to be clarified.

Finally, the in vivo administration of LP-211, a selective 5-HT_7_ receptor agonist with drug-like properties [87], rescued object recognition memory and reduced stereotyped marble burying behavior in *Fmr1* KO mice (Figure 1B) [72]. Accordingly, the administration of LP-211 reduced repetitive self-grooming behavior in *Fmr1* KO mice [73]. These data together suggest that 5-HT_7_ receptor agonists might be further studied in view of clinical studies on Fragile X patients with cognitive impairment and autistic behavior.

## 4. Drugs Acting on Distinct 5-HT_2_ Receptor Subtypes Rescued Synaptic Plasticity and Learning in *Fmr1* KO Mice

The activation of 5-HT_2_ receptors was found to inhibit NMDA-receptor-induced LTP in wild-type rat visual cortex by a mechanism involving phospholipase C signaling and GABAergic interneurons; the subtype of 5-HT_2_ receptors was not identified [88]. Later work investigated the effects of distinct 5-HT_2_ receptor subtypes (5-HT_2A,_ 5-HT_2B_ and 5-HT_2C_) on NMDA-receptor-mediated synaptic plasticity in wild-type and *Fmr1* KO mice. In mouse anterior cingulate cortex (a limbic brain area involved in attention, reward, decision-making and emotions), the inhibition of 5-HT_2A_ receptors enhanced NMDA-receptor-induced LTP in wild-type animals but not in *Fmr1* KO mice, in which LTP had a small amplitude and was not potentiated by 5-HT_2A_ antagonists. The effect of 5-HT_2A_ antagonists in wild-type mice relied on a post-synaptic mechanism; since the expression level of 5-HT_2_ receptors in the anterior cingulate cortex of *Fmr1* KO mice was not modified with respect to wild-type mice, the authors suggest a disruption of receptor functioning [89]. Another work instead shows that in *Fmr1* KO mice, a 5-HT_2A_ receptor antagonist rescued alterations in the membrane trafficking of GluA1 AMPA receptor subunits and LTP defects in the hippocampus and brain cortex, as well as learning deficits evaluated by the fear conditioning protocol and Y maze test [68]. No rescue effect was observed after the selective modulation of 5-HT_2C_ receptors, nor when all 5-HT_2_ receptor subtypes were activated by a non-selective 5-HT_2_ receptor agonist. The same work shows that a recovery of synaptic plasticity and learning in *Fmr1* KO mice was observed after the application of either a 5-HT_2A_ receptor antagonist or a 5-HT_2B_ receptor agonist, a D1 receptor agonist and a D2 receptor antagonist; interestingly, a combination of 5-HT and DA agonists and antagonists at very low doses synergistically rescued synaptic plasticity and learning, suggesting that therapy with a drug cocktail at low doses might be effective, while reducing adverse effects.

These data (Table 1) indicate that either the blockade of 5-HT_2A_ receptors or activation of 5-HT_2B_ receptors corrects reduced LTP and learning deficits in *Fmr1* KO mice.

## 5. Activation of 5-HT_1A_ Receptors Corrected Abnormal Phenotypes in Mouse and Drosophila Models of Fragile X Syndrome

In addition to 5-HT_2A_ receptors, 5-HT_1A_ receptors also inhibited NMDA-mediated synaptic responses and LTP in rat primary visual cortexes [90,91]. The effects of 5-HT_1A_ receptors on LTP have not been tested in *Fmr1* KO mice; it would be interesting to check if the blockade of 5-HT_1A_ receptors might rescue the reduced LTP in *Fmr1* KO mice.

On the other side, the activation (rather than blockade) of 5-HT_1A_ receptors is able to prevent seizures and rescue behavior in *Fmr1* KO mice (Table 1). The aminotetralin (S)-5-(2′-fluorophenyl)-N,N-dimethyl-1,2,3,4-tetrahydronaphthalen-2-amine (FPT), a partial agonist of the 5-HT_1A_, 5-HT_2C_ and 5-HT_7_ receptors and a full agonist of the 5-HT_1B_, and 5-HT_1D_ receptors, reduced repetitive behaviors and increased social attitude in wild-type mice [92]. In *Fmr1* KO mice, the in vivo administration of FPT completely prevented audiogenic seizures, exerted anxiolytic effects and increased social interactions [63]. Accordingly, the in vivo administration of NLX-101, a selective 5-HT_1A_-biased agonist, reduced audiogenic seizures in *Fmr1* KO mice; the authors made the hypothesis that 5-HT_1A_ receptors located in the inferior colliculus inhibit hyperactive neurons responsible for auditory hypersensitivity and generating audiogenic seizures [65].

In *Fmr1* KO mice, similar to Fragile X patients, neuronal hyperexcitability is also present in the somatosensory and auditory brain cortexes, and can be evidenced by altered electroencephalogram activity, with an abnormal increase in gamma waves (30–80 Hz) being seen. The in vivo administration of FPT corrected abnormal electroencephalogram activity in the somatosensory cortex and exerted different sex-specific effects in the auditory cortex of adult *Fmr1* KO mice [64].

In line with the above cited results, a reduced 5-HT_1A_ receptor expression was recently described in the brain of juvenile (not adult) *Fmr1* KO mice at the age of highest seizure susceptibility: at this age, the administration of NLX-112, a selective 5-HT_1A_ receptor agonist, effectively prevented seizures [66]. The authors also suggested that antiepileptic treatment with a 5-HT_1A_ receptor agonist should be started in young Fragile X patients.

A very recent study shows that a drosophila model of Fragile X Syndrome displayed an abnormally increased number of mitochondria and oxidative hyperactivity in the neurons of the neuromuscular junction, together with a reduced locomotor activity; these malfunctions were corrected by treatment with eltoprazine, a selective 5-HT_1A_ receptor agonist [67]. Abnormal mitochondrial functions were revealed in human Fragile X-associated tremor ataxia syndrome (the pre-mutation condition) [93] and in *Fmr1* KO mice [94,95,96]. It would be very interesting to check if 5-HT_1A_ receptor agonists might also rescue mitochondrial deficits in *Fmr1* KO mice and in human Fragile X patients.

## 6. The Blockade of 5-HT_5A_ Receptors Improved Behavior and Memory in a Rat Model of Fragile X Syndrome

Another possible target for Fragile X Syndrome therapy is the 5-HT_5_ receptor (Table 1). A recent study shows that *Fmr1* KO rats displayed hyperactivity, abnormal sensory motor gating (as measured by the prepulse inhibition of startle protocol) and memory deficits (evaluated by the Novel Object Recognition test), and all these abnormal phenotypes were rescued by the oral administration of ASP5736, a selective 5-HT_5A_ receptor antagonist [69]. As a possible action mechanism, the authors propose that the blockade of 5-HT_5A_ receptors located on GABAergic interneurons in the ventral tegmental area might induce disinhibition and enhance GABA release onto dopaminergic neurons. Interestingly, the same authors had previously shown that the administration of ASP5736 also rescued cognitive deficits in aged rats [97] and in animal models of schizophrenia [98,99], pointing out 5-HT_5_ receptor antagonists as a new potential therapy tool in different conditions with cognitive impairment. The effects of drugs modulating serotonergic transmission in Fragile X patients and animal models are summarized in Table 1; Table 2 illustrates the main features of the serotonin receptors rescuing abnormal phenotypes in Fragile X animal models.

## 7. Conclusions

An overall enhancement of serotonergic transmission with SSRIs improves mood disorders and language deficits in Fragile X patients, and is already successfully used in therapy. Studies on Fragile X animal models further suggest that the selective modulation of the 5-HT_1A_, 5-HT_2A_, 5-HT_2B_, 5-HT_5A_ and 5-HT_7_ receptors might be used to specifically correct other symptoms: these receptors, each having a different brain localization and activating different signaling mechanisms (Table 2), were shown to correct distinct malfunctions in Fragile X animal models. 5-HT_1A_ agonists very effectively prevented seizures in *Fmr1* KO mice, and therefore might be envisaged to treat epileptic Fragile X patients. 5-HT_2A_ antagonists and 5-HT_2B_ agonists rescued LTP and learning in *Fmr1* KO mice, and thus might improve cognition. 5-HT_5A_ antagonists reduced hyperactivity and improved cognition in *Fmr1* KO rats, and thus might be used in Fragile X patients with concomitant attention deficit and hyperactivity disorder (ADHD). 5-HT_7_ agonists rescued synaptic plasticity, learning deficit and stereotyped behavior in *Fmr1* KO mice, and therefore might be proposed to treat Fragile X patients with cognitive impairment and autistic features.

In conclusion, the treatment of Fragile X patients with SSRIs might be associated with drugs selectively acting on specific 5-HT receptor subtypes, depending on the patient’s symptoms. Future studies will better clarify the role of different 5-HT receptor subtypes in the disease in order to refine differential strategies for pharmacological intervention.

The development of new pharmacological strategies for Fragile X Syndrome is particularly important because we still lack an effective treatment, although several promising drug candidates are under investigation. Past clinical trials targeting metabotropic glutamate receptors have shown no significant improvements on Fragile X patients [100,101,102]. Other strategies aiming to correct malfunctions in brain GABAergic transmission have given disappointing results: the administration of ganaxolone, a neurosteroid positively modulating GABA_A_ receptors, did not induce any significant improvement in anxiety, attention or hyperactivity in children with Fragile X Syndrome [103], and treatment with arbaclofen, a GABA_B_ receptor agonist, did not improve social behavior [104].

On the other hand, other studies show promising results. A phase 2 clinical trial was conducted on 30 male Fragile X patients who were administered an inhibitor of phosphodiesterase 4 (PDE4) in order to enhance cAMP signaling (which is depressed in Fragile X Syndrome); results from this study show an improvement in cognition, language and daily functioning [105].

A combined treatment of lovastatin and minocycline was recently shown to reduce abnormal cortical excitability in Fragile X patients [106].

Another candidate drug for Fragile X therapy is metformin, based on the observation that the absence of FMRP leads to the increased brain production of Insulin-like Growth Factor 1 (IGF-1) [107]. A recent report from a clinical trial shows that metformin administration to young Fragile X patients (6–25 years old) prevented a decline in cognitive development and adaptive functioning, although this result needs to be confirmed in a large sample of patients [108].

## 8. Future Directions

Clinical studies might be designed to test the effects of drugs selectively targeting 5-HT receptor subtypes on Fragile X patients, alone or in association with SSRI treatment. Some of these drugs are already used in therapy, and thus their off-label use in Fragile X Syndrome might be envisaged.

Buspirone, a partial agonist of 5-HT_1A_ receptors, is used as an antidepressant and anxiolytic [109]. The atypical antipsychotic aripiprazole, besides activating the D2 and D3 dopamine receptors, has a partial agonist activity at 5-HT_1A_ receptors and an antagonist activity at 5-HT_2A_ receptors [110] and is at present used to treat Fragile X patients with aggressive and self-injurious behavior [111]. Pimavanserin, a new generation 5-HT_2A_ selective antagonist, has been clinically tested as an antipsychotic in Parkinson’s disease and schizophrenia; its pharmacokinetic properties, safety and tolerability have been characterized in humans [112]. New selective 5-HT_7_ receptor agonists with drug-like properties have been synthetized and tested in pre-clinical studies on *Fmr1* KO mice [71,113]. Interestingly, a set of arylpiperazine derivatives with a mixed 5-HT_1A_/5-HT_7_ agonist and 5-HT_2A_ antagonist activity, a good in vitro metabolic stability, drug-like properties and the ability to cross the blood–brain barrier have recently been proposed as a potential tool to treat autism spectrum disorders [114]; based on their mixed pharmacological properties, we suggest that these compounds might be tested in preclinical studies on animal models of Fragile X Syndrome.

## Figures and Tables

**Table 2:** Main features of 5-HT_1A_, 5-HT_2A_, 5-HT_2B_, 5-HT_5A_ and 5-HT_7_ receptors for serotonin.

Receptor	Brain Localization (Rodent; Human)	Transduction Mechanism	Agonists	Antagonists	References
5-HT_1A_	Limbic areas; hippocampus; raphe nuclei	Inhibition of adenylate cyclase	8-OH DPAT	WAY100635	[4]
5-HT_2A_	Cerebral cortex; olfactory bulb; brainstem nuclei	Stimulation of phospholipase C	DOI	Ketanserin MDL100907	[4]
5-HT_2B_	Cerebellum; lateral septum; hypothalamus; amygdala	Stimulation of phospholipase C	BW723C86	SB200646 SB204741	[4]
5-HT_5A_	Hippocampus; frontal cortex; raphe nuclei	Inhibition of adenylate cyclase	5-CT 8-OH DPAT	ASP5736	[11] [69]
5-HT_7_	Thalamus; hypothalamus; hippocampus	Stimulation of adenylate cyclase	5-CT 8-OH DPAT LP-211	SB269970 SB656104	[16]; [87]
